# Long noncoding RNA TMEM147-AS1 serves as a microRNA-326 sponge to aggravate the malignancy of gastric cancer by upregulating SMAD5

**DOI:** 10.32604/or.2022.03568

**Published:** 2022-08-31

**Authors:** XUFU QIN, ZIYE JIANG, YONGCUI ZHU, HONGPENG XUE, CHENGQUN WEI

**Affiliations:** 1 Second Department of Gastroenterology, Heilongjiang Provincial Hospital, Harbin, 150001, China; 2 Department of General Medicine, Heilongjiang Provincial Hospital, Harbin, 150001, China

**Keywords:** TMEM147-AS1, Gastric cancer, ceRNA, miRNA sponge

## Abstract

The abnormal expression of long noncoding RNAs (lncRNAs) is frequently observed in gastric cancer (GC) and considered an important driving force in GC progression. However, little is known regarding the involvement of TMEM147-AS1 in GC. Therefore, we examined TMEM147-AS1 expression in GC and determined its prognostic value. In addition, TMEM147-AS1 expression was depleted to identify the functional changes in response to TMEM147-AS1 deficiency. Using the cancer genome atlas dataset and our own cohort, we identified a strong expression of TMEM147-AS1 in GC. Increased TMEM147-AS1 levels in GC showed a significant association with poor prognosis. TMEM147-AS1 interference resulted in the inhibition of GC cell proliferation, colony-forming, migration, and invasion *in vitro*. Additionally, depletion of TMEM147-AS1 restricted the growth of GC cells *in vivo*. Mechanistically, TMEM147-AS1 functioned as a microRNA-326 (miR-326) sponge. Furthermore, SMAD family member 5 (SMAD5) was experimentally validated as the functional effector of miR-326. TMEM147-AS1 was demonstrated to sequester miR-326 away from SMAD5; consequently, knocking down TMEM147-AS1 downregulated SMAD5 levels in GC cells. The functional suppression of miR-326 or reintroduction of SMAD5 effectively reversed the attenuated behavior of GC cells caused by TMEM147-AS1 downregulation. In summary, TMEM147-AS1 exhibits tumorigenic activities in GC, which is likely the result of an altered miR-326/SMAD5 axis. Therefore, targeting TMEM147-AS1/miR-326/SMAD5 may represent a target for the treatment of GC.

## Introduction

Gastric cancer (GC) is the fifth most common form of human cancer and is the third most common cause of tumor-associated deaths globally [1]. Annually, approximately 950,000 new GC cases are diagnosed worldwide and over 720,000 patients die from this disease [2]. Currently, therapeutic strategies for GC include surgery and adjuvant chemotherapy; however, these treatments have drawbacks such as toxicity, chemoresistance, eventual relapse, and metastasis [3]. Although significant advances have been made regarding diagnostic and curative methods, the poor clinical efficiency in patients with GC remains a significant problem and should be addressed [4]. Over the last few decades, considerable progress has been made toward the understanding of GC pathogenesis; however, there remain limitations in the application of these research findings to new anticancer treatments [5,6]. Therefore, elucidating the mechanisms associated with gastric carcinogenesis and progression may help design promising anticancer therapies.

Long noncoding RNAs (lncRNAs) belong to a group of transcripts containing over 200 nucleotides in length but are deficient in protein-coding ability [7]. Reportedly, lncRNAs exert important modulatory activities in cell physiology and pathology, although they were previously considered “dark matter” or “noise” in the transcriptome [8]. Regarding GC, compelling studies have demonstrated that a considerable number of lncRNAs are differentially expressed and their dysregulation contributes to the malignant characteristics of GC [9–11].

MicroRNAs (miRNAs) are an emerging family of endogenous, single-stranded, noncoding transcripts of 17–23 nucleotides in length [12]. They can recognize and bind to the 3′-untranslated regions of target genes through base pairing, thereby degrading mRNAs or inhibiting translation [13]. MiRNAs are intimately involved in the control of various behaviors underlying cancer etiology and development [14]. Recently, the competitive endogenous RNA (ceRNA) theory was introduced, which postulated that lncRNAs, by sequestering miRNAs, can reduce the amount of miRNAs available for downstream targets, which in turn prevents the target mRNAs from being degraded by miRNAs [14]. This mechanism, known as the ceRNA pathway, includes lncRNAs, miRNAs, and mRNA, which may contribute to GC therapy.

Through The Cancer Genome Atlas (TCGA), we identified that TMEM147-AS1 was overexpressed in GC, which suggests its role in GC progression. As a novel tumor-associated lncRNA, the role of TMEM147-AS1 in GC progression remains largely unknown. Therefore, we evaluated TMEM147-AS1 expression in GC and determined its prognostic value. In addition, TMEM147-AS1 expression was depleted to examine the functional changes in GC cells in response to TMEM147-AS1 deficiency. Overall, our study illuminates a novel ceRNA pathway in GC comprising TMEM147-AS1, miR-326, and SMAD5.

## Materials and Methods

### Clinical tissues and cells

This study was approved by the Ethics Committee of Heilongjiang Provincial Hospital and was conducted in accordance with the ethical principles of the Declaration of Helsinki. Upon receiving signed informed consent, GC tissues and nontumor gastric mucosa were collected from 49 patients at our hospital. No patients had received any type of anticancer therapy. After obtaining the tissues during surgery, they were stored in liquid nitrogen until further use.

The human gastric epithelial cell line GES-1 (Beyotime; Shanghai, China) and GC cell lines SNU-1 and HGC27 (National Collection of Authenticated Cell Cultures; Shanghai, China) were grown in Roswell Park Memorial Institute Medium-1640 (both from Gibco; Thermo Fisher Scientific, Inc., Waltham, MA, USA) supplemented with 10% fetal bovine serum (FBS). F12K medium (Gibco; Thermo Fisher Scientific) was used for the GC cell line AGS (National Collection of Authenticated Cell Cultures). All basal media were supplemented with 10% FBS and 1% penicillin–streptomycin (Gibco; Thermo Fisher Scientific, Inc.). All cells were grown in a humidified incubator at 37°C under 5% CO_2_.

### Transfection

GenePharma Co., Ltd. (Shanghai, China) constructed the small interfering RNAs targeting TMEM147-AS1 (si-TMEM147-AS1s). The nontargeted siRNA was used as the control. MiR-326 mimic/inhibitor, negative control (NC) miRNA mimic (miR-NC), and NC inhibitor was obtained from RiboBio (Guangzhou, China). The pcDNA-SMAD5 (pc-SMAD5) plasmid was constructed by GeneChem (Shanghai, China) to overexpress SMAD5. Cells were seeded into 6-well plates a day before transfection. All transfections were performed using Lipofectamine 2000 (Invitrogen; Thermo Fisher Scientific, Inc., Carlsbad, CA, USA), and quantitative real-time polymerase chain reaction (qRT-PCR) was used for assessing the transfection efficiency.

### qRT-PCR

Total RNA was extracted using TRIzol (Invitrogen; Thermo Fisher Scientific, Inc.). Complementary DNA was synthesized from mRNA using the PrimeScript^™^ RT reagent Kit (TaKaRa; Dalian, China), whereas the miScript Reverse Transcription kit (Qiagen GmbH, Hilden, Germany) was used for reverse transcription of miRNA. To quantify TMEM147-AS1 and SMAD5, PCR amplification was performed using TB Green® Premix Ex Taq^™^ (TaKaRa) with GAPDH as the reference gene. To assess miR-326 levels, the miScript SYBR Green PCR kit (Qiagen GmbH) was used for quantitative PCR with U6 small nuclear RNA as the reference gene. The 2^−ΔΔCq^ method was used for data analysis.

### Cell counting kit-8 (CCK-8) assay

Transfected cells at a concentration of 3,000 cells per well were seeded into 96-well plates. Prior to the addition of 10 µl of cell counting kit-8 reagent (Beyotime), the cells were cultured for different time periods. Following 2 h of incubation at 37°C, a microplate reader was used to detect the absorbance at a wavelength of 450 nm.

### Colony formation assay

Transfected cells were harvested at 24 h post-transfection. Cell suspension was prepared, and seeded into 6-well plates. Each well covered 2 ml cell suspension containing 500 cells. After culturing for 2 weeks, the colonies were fixed applying 4% paraformaldehyde, and subjected for coloration with 0.1% crystal violet. After washing and drying, the visible colonies were photographed and counted.

### Transwell migration and invasion assays

Transwell inserts equipped with an 8.0-μm pore polycarbonate membrane (BD Biosciences, Franklin Lakes, NJ, USA) were used for the assay. For the migration assay, the upper chambers were loaded with 5 × 10^4^ cells in 200 µl of serum-free medium, and 600 µl of complete culture medium was added to the lower chambers. After 24 h of incubation at 37°C, the nonmigrated cells were cleaned with a cotton swab. The cells were fixed in ethanol and stained with 0.1% crystal violet. For the invasion assay, the same experimental procedures were followed, except the inserts were precoated with Matrigel (BD Biosciences). Upon termination, the number of stained cells was counted in five randomly selected fields.

### Xenograft mice assay in vivo

All animal experiments were approved by the Animal Care and Use Committee of Heilongjiang Provincial Hospital. Short hairpin RNA (shRNA) targeting TMEM147-AS1 (sh-TMEM147-AS1) and nontargeted shRNA (sh-NC; both from GenePharma) were inserted into lentivirus vector. After transfecting into 293T cells for 48 h, the lentiviruses were collected and employ for infecting HGC27 cells. HGC27 cells with stable transfection were chosen by cultivating with puromycin. Male BALB/c nude mice (Vital River Laboratory Animal Technology Co., Ltd., Beijing, China), aged 4–6 weeks, were subcutaneously injected with sh-TMEM147-AS1 or sh-NC stable transected-HGC27 cells. Weekly, the width and length of tumors was recorded, and mice were euthanized 4 weeks after cell injection. Tumor xenografts were excised, weighted and photographed. The volume was detected with the formula: Volume = 0.50 × length × width^2^.

### Nuclear/cytoplasmic fractionation

GC cells were collected and lysed to isolate nuclear and cytoplasmic fractions using the Cytoplasmic and Nuclear RNA Purification Kit (Norgen, Thorold, ON, Canada). TRIzol was used to isolate RNA from both fractions, and qRT-PCR was performed to assess relative TMEM147-AS1 levels.

### Bioinformatics tools

LncBase Experimental v.2 (http://diana.imis.athena-innovation.gr/DianaTools/index.php?r=lncExperimental/index) and starBase 3.0 (https://starbase.sysu.edu.cn/) revealed the target binding between TMEM147-AS1 and miR-326. Bioinformatics tools, starBase 3.0, miRDB (http://mirdb.org/), and TargetScan (http://www.targetscan.org/), were used to predict the target of miR-326. TCGA dataset was used to examine the expression of TMEM147-AS1 in GC. Also, the expression correlation among TMEM147-AS1, miR-326 and SMAD5 in GC tissue samples from TCGA dataset was explored.

### Luciferase reporter assay

TMEM147-AS1 and SMAD5 fragments harboring the miR-326 wild-type (wt) target site were amplified and inserted into pmirGLO vectors to generate the wt-TMEM147-AS1 and wt-SMAD5 vectors. Simultaneously, the same method was used to prepare luciferase reporter vectors carrying the mutant (mut) targets, mut-TMEM147-AS1 and mut-SMAD5. GC cells were seeded into 24-well plates and transfected with wt or mut vectors in the presence of miR-326 mimic or miR-NC. After 48 h, the luciferase activity of the transfected cells was measured using the Dual-Luciferase Reporter Assay system (Promega, Madison, WI, USA).

### RNA immunoprecipitation

The binding interaction among TMEM147-AS1, miR-326, and SMAD5 was determined using the Magna RIP RNA Binding Protein Immunoprecipitation Kit (Merck Millipore, Darmstadt, Germany). In brief, whole GC cell lysates were obtained by incubating cells with RNA immunoprecipitation (RIP) lysis buffer, followed by mixing with magnetic beads coupled to anti-Argonaute2 (Ago2) antibodies or immunoglobulin G control (Millipore). Next, the magnetic beads were collected and rinsed with RIP buffer. After incubating the protein with proteinase K, the coprecipitated RNA was extracted and analyzed using qRT-PCR.

### Western blotting

Total protein was extracted and quantified using radioimmunoprecipitation assay buffer and the BCA Kit (both from KeyGen; Nanjing, China), respectively. Equal amounts of protein were separated through electrophoresis on 10% SDS-PAGE gels. After transferring to polyvinylidene fluoride membranes and blocking with 5% nonfat milk, the membranes were incubated with primary antibodies for SMAD5 (ab40771) or GAPDH (ab128915; both from Abcam, Cambridge, MA, USA) at 4°C overnight. Incubation with horseradish peroxidase-coupled secondary antibodies (ab205718; Abcam) was performed, and the BeyoECL Moon Kit (Beyotime) was used to generate the chemiluminescent signal.

### Statistical analysis

The results from three biological replicates for each experiment were expressed as means ± standard deviations. Comparisons between groups were performed using Student’s *t*-test (2 groups) or one-way analysis of variance (ANOVA; ≥3 groups). Tukey’s post hoc test was used following ANOVA. Survival curves were plotted following the Kaplan–Meier method and analyzed using the log-rank test. Gene expression analysis was performed using Pearson’s correlation analysis. *p* < 0.05 was considered statistically significant.

## Results

### Elevated TMEM147-AS1 levels in GC

**Figure 1:**
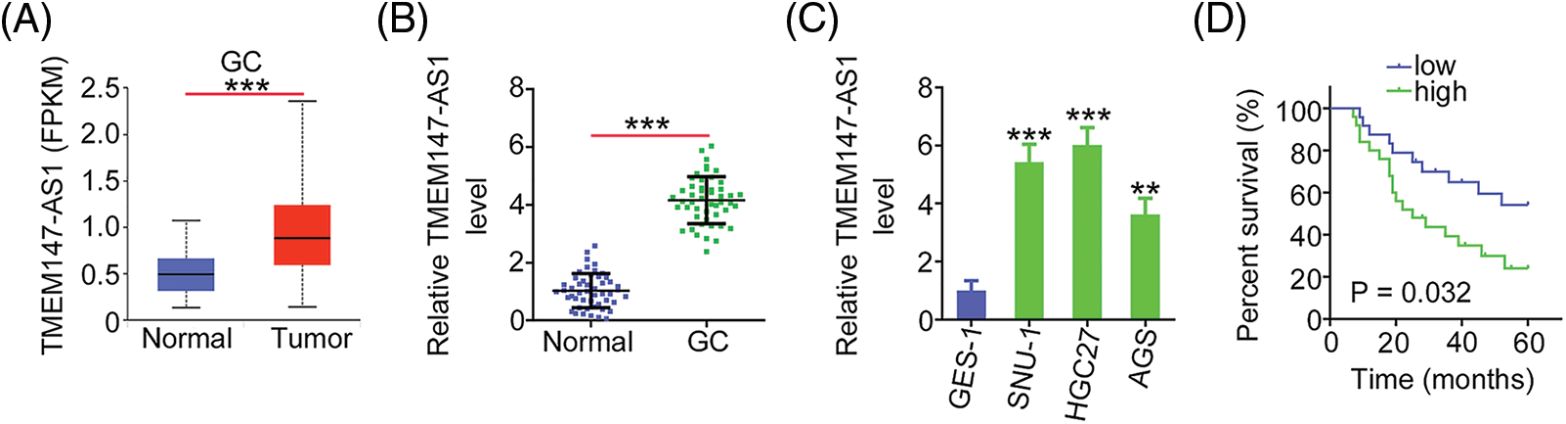
TMEM147-AS1 is overexpressed in GC. (A) TMEM147-AS1 level in GC was analysed via TCGA. ***p < 0.001 (n = 3) compared with normal. (B) qRT-PCR illuminated the level of TMEM147-AS1 in GC tissues and non-tumor gastric mucosa. ***p < 0.001 (n = 3) compared with normal. (C) Level of TMEM147-AS1 was detected in three GC cell lines. ***p < 0.001 compared with GES-1. **p < 0.01 (n = 3) compared with GES-1. (D) Kaplan–Meier method was realized for determining the overall survival in GC patients with high or low TMEM147-AS1 level. *p* = 0.0032 (n = 3).

The expression of TMEM147-AS1 in GC was initially evaluated using TCGA database. Fig. 1A shows that compared with normal tissues, the levels of TMEM147-AS1 considerably increased in GC tissues. Next, we compared TMEM147-AS1 expression between GC tissues and nontumor gastric mucosa obtained from our cohort. GC tissues exhibited significantly higher levels of TMEM147-AS1 than normal gastric mucosa (Fig. 1B). Furthermore, the overexpression of TMEM147-AS1 was noted in the GC cell lines (Fig. 1C). Using the median value of TMEM147-AS1 in GC tissues as the cutoff, the participants were categorized into TMEM147-AS1-low or TMEM147-AS1-high groups. A high TMEM147-AS1 level was associated with an overall survival poorer than that of those with a low TMEM147-AS1 level (Fig. 1D). Thus, TMEM147-AS1 may be an important modulator of GC progression.

### TMEM147-AS1 interference executes anticarcinogenic activities in GC

**Figure 2:**
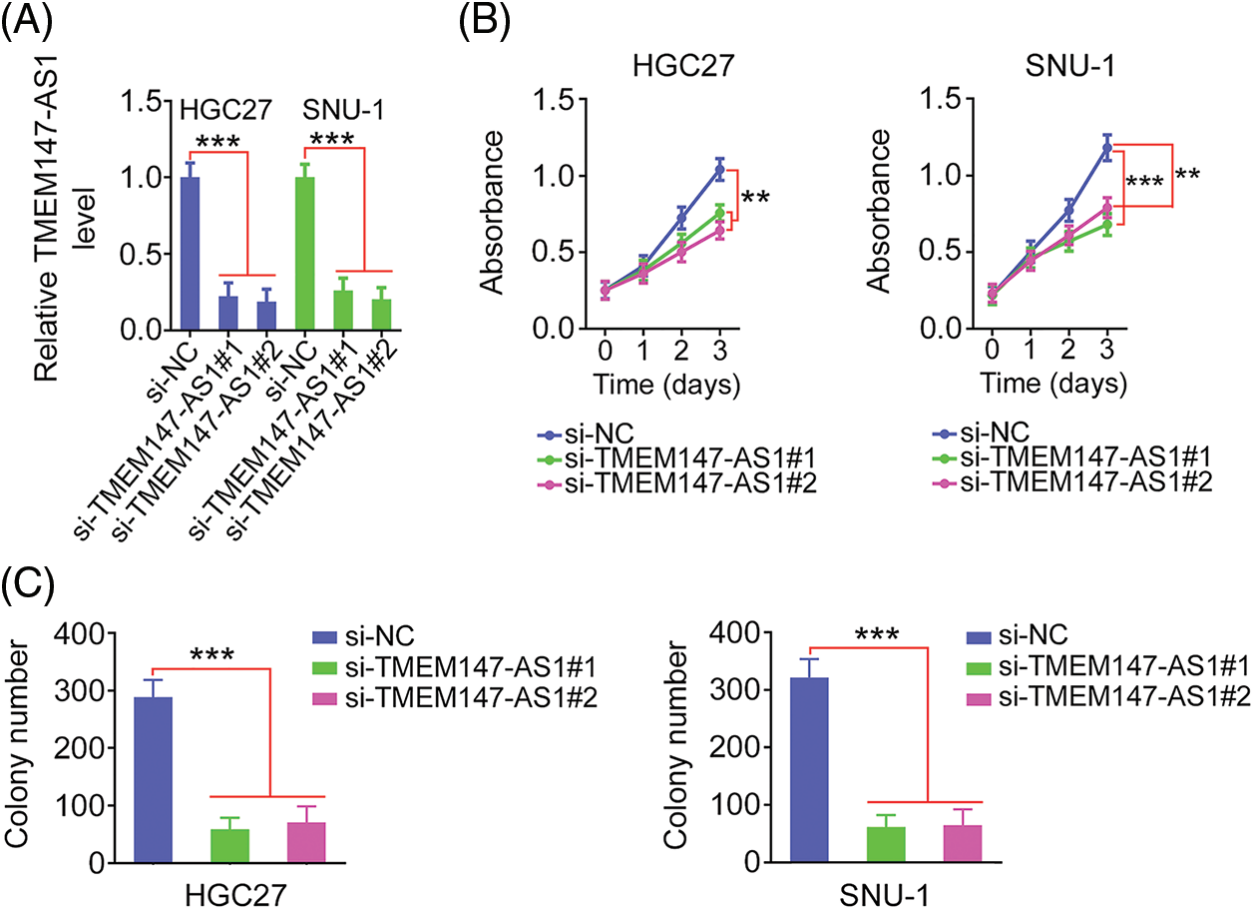
TMEM147-AS1 deficient exerts repressing activity on GC cell phenotype. (A) qRT-PCR was utilized for the measurement of TMEM147-AS1 in GC cells when were transfected with si-TMEM147-AS1 or si-NC. ***p < 0.001 (n = 3) compared with si-NC. (B) CCK-8 assay was conducted in GC cells after TMEM147-AS1 depletion. ***p < 0.001 (n = 3) compared with si-NC. **p < 0.01 (n = 3) compared with si-NC. (C) The colony formation of si-TMEM147-AS1-expressing GC cells was quantified via colony formation assay. ***p < 0.001 (n = 3) compared with si-NC.

**Figure 3:**
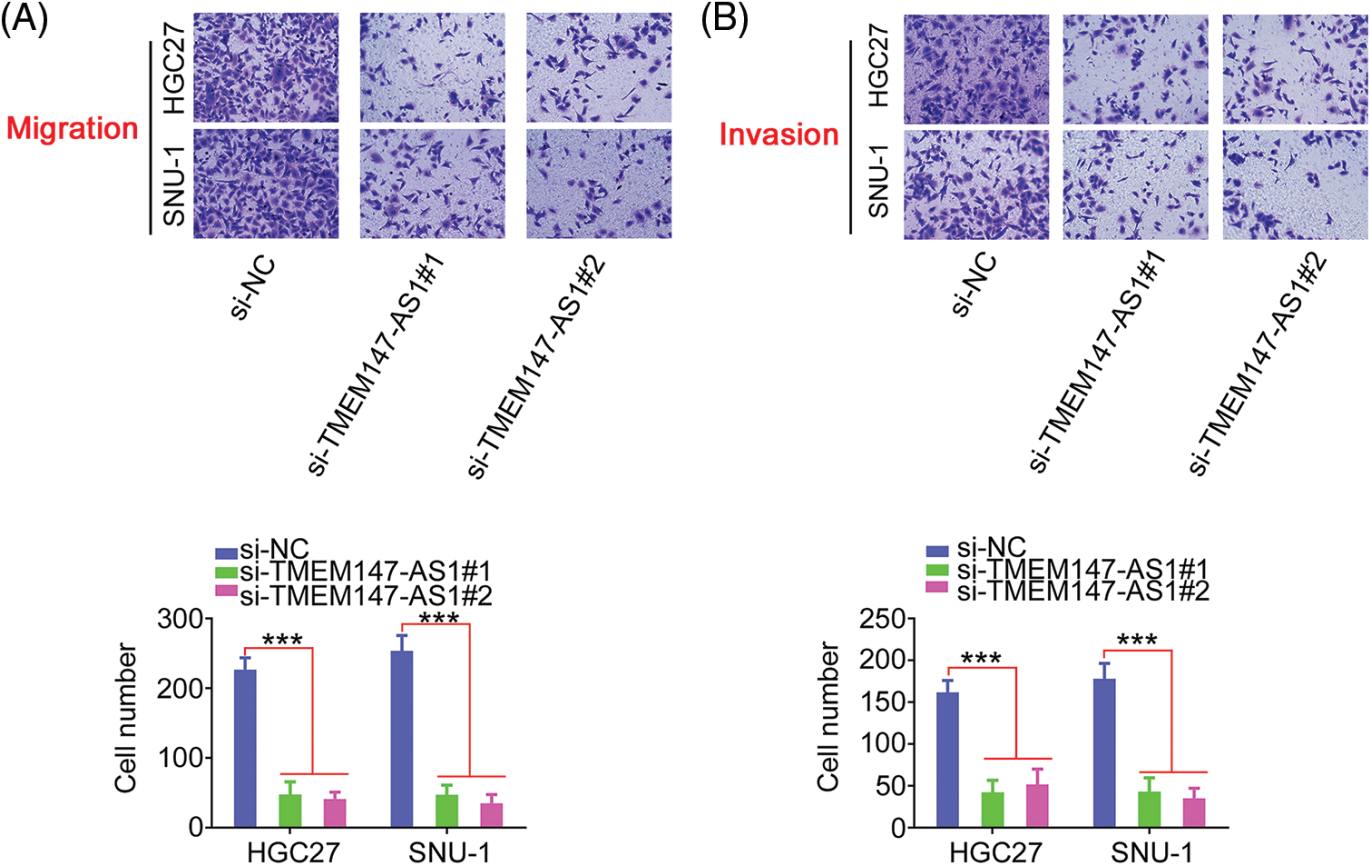
TMEM147-AS1 depletion inhibits GC cell motility. (A, B) The migration and invasion of TMEM147-AS1 silenced-GC cells was tested via Transwell assay. 100× magnification. ***p < 0.001 (n = 3) compared with si-NC.

Given the elevated TMEM147-AS1 levels in GC, we examined the putative function of TMEM147-AS1 in GC. The overexpression of TMEM147-AS1 was most pronounced in HGC27 and SNU-1 cells; thus, they were used for the subsequent experiments. We used two siRNAs, si-TMEM147-AS1#1 and si-TMEM147-AS1#2, to avert off-target effects. TMEM147-AS1 was depleted by transfecting with si-TMEM147-AS1 (Fig. 2A), which was confirmed through qRT-PCR. Based on the results of the CCK-8 and colony formation assays, cell proliferation and colony formation was noted to be attenuated in GC cells following TMEM147-AS1 ablation (Figs. 2B and 2C). Moreover, the migration and invasion capacities were strikingly impeded in TMEM147-AS1-deficient GC cells (Figs. 3A and 3B). Therefore, the loss of TMEM147-AS1 disrupts GC progression.

### TMEM147-AS1 sequesters miR-326 in GC

**Figure 4:**
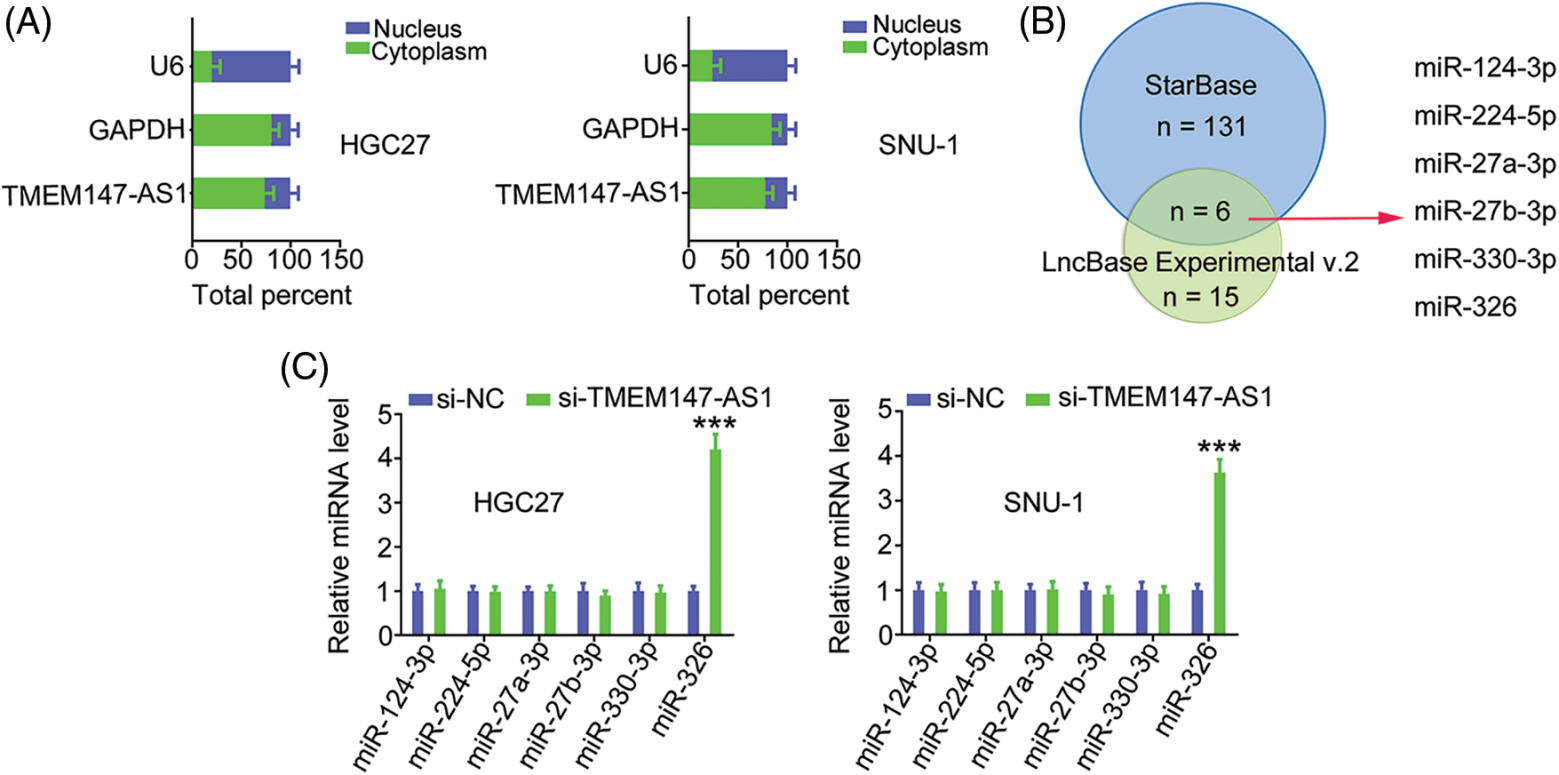
TMEM147-AS1 is a as a cytoplasm-bound lncRNA and may sponge miR-326 in GC. (A) The nuclear and cytoplasmic fractions were isolated, and subjected to qRT-PCR for assessing TMEM147-AS1 location. (B) The targets of TMEM147-AS1 predicted by LncBase Experimental v.2 and starBase 3.0. (C) Expression of 6 candidates in TMEM147-AS1 depleted-GC cells was tested employing qRT-PCR. ***p < 0.001 (n = 3) compared with si-NC.

Having confirmed the effects of TMEM147-AS1 on GC progression, we examined the mechanisms of TMEM147-AS1-induced regulation. First, we used nuclear/cytoplasmic fractionation to identify TMEM147-AS1 as a cytoplasm-bound lncRNA (Fig. 4A). The result suggests that TMEM147-AS1 is a ceRNA that can decoy certain miRNAs to modulate gene expression. Using bioinformatics tools, six overlapping miRNAs (Fig. 4B) were selected for further study. We measured the levels of these six candidates in TMEM147-AS1-silenced GC cells. MiR-326 level was notably increased in GC cells when TMEM147-AS1 was knocked down, whereas the expression of the other five miRNAs remained unaffected (Fig. 4C).

**Figure 5:**
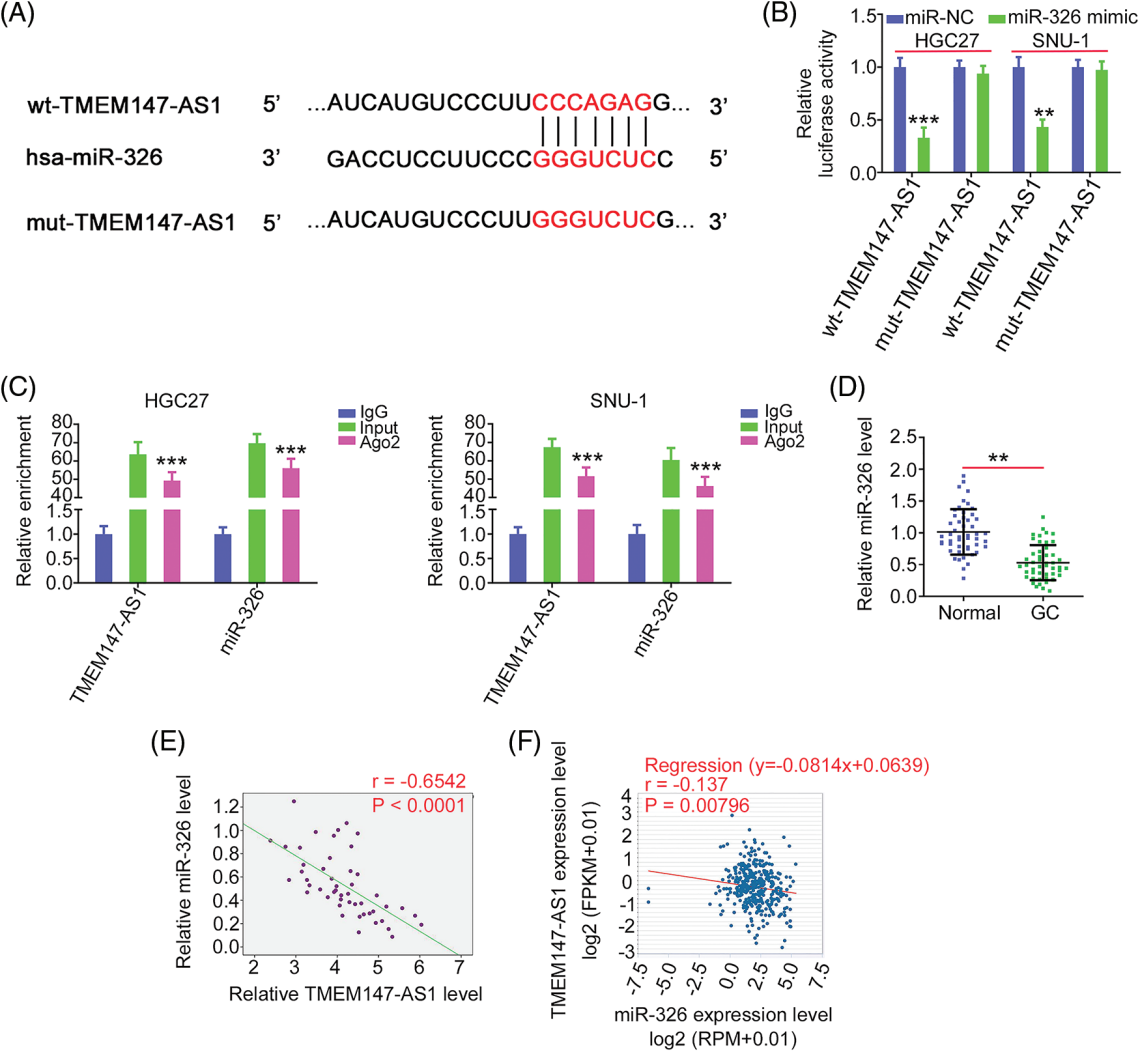
miR-326 is decoyed by TMEM147-AS1 in GC. (A) Schematic representation of the binding sequences between TMEM147-AS1 and miR-326. (B) The luciferase activity driven by wt-TMEM147-AS1 or mut-TMEM147-AS1 was quantified in GC cells in the presence of miR-326 mimic or miR-NC. ***p < 0.001 (n = 3) compared with miR-NC. **p < 0.01 (n = 3) compared with miR-NC. (C) RIP assay applying Ago2 antibody certified the enrichment of TMEM147-AS1 and miR-326. ***p < 0.001 (n = 3) compared with IgG. (D) qRT-PCR detected the level of miR-326 in GC tissues. **p < 0.01 (n = 3) compared with Normal. (E) Pearson’s correlation analysis was conducted to confirm the relation between TMEM147-AS1 and miR-326 levels in GC tissues from our own cohort. *p* < 0.0001 (n = 3). (F) Pearson’s correlation analysis was conducted to confirm the relation between TMEM147-AS1 and miR-326 levels in GC samples from TCGA database. *p* = 0.00796 (n = 3).

MiR-326 contains a TMEM147-AS1 complementary binding site (Fig. 5A); therefore, we performed a luciferase reporter assay to verify binding. MiR-326 was sufficient to reduce the luciferase activity of wt-TMEM147-AS1, which largely recovered when the binding sequences were mutated (Fig. 5B). As shown in Fig. 5C, TMEM147-AS1 and miR-326 were markedly enriched by the Ago2 antibody, suggesting that they are recruited to the same RNA-induced silencing complex. In addition, downregulated miR-326 in GC tissues (Fig. 5D) exhibited an inverse expression relationship with TMEM147-AS1 (Fig. 5E), which is consistent with the results obtained from the correlation analyses of the GC samples from TCGA database (Fig. 5F). In summary, TMEM147-AS1 acts as a sponge for miR-326 in GC.

### MiR-326 directly targets SMAD5

**Figure 6:**
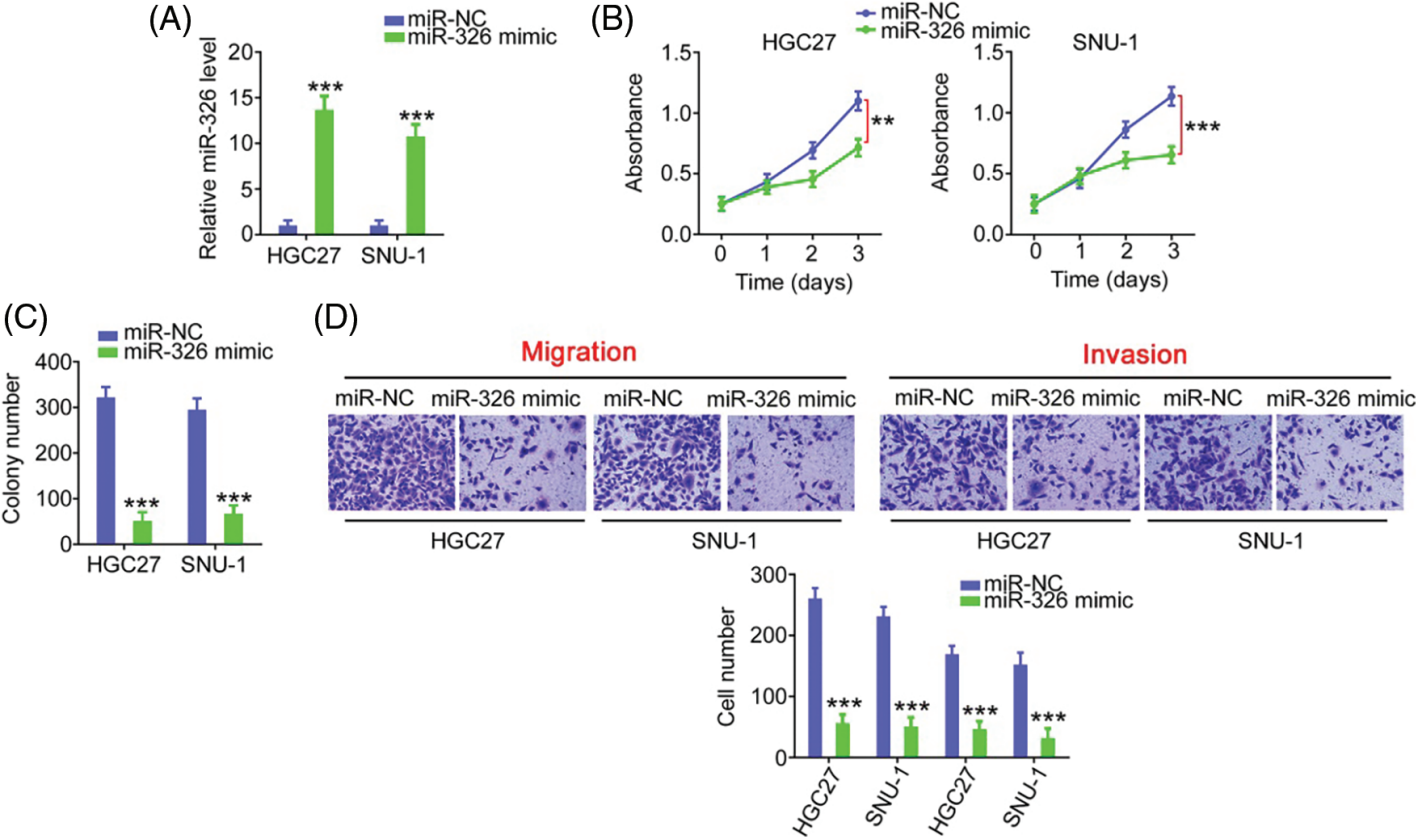
MiR-326 overexpression suppresses GC cell proliferation and motility. (A) qRT-PCR detected miR-326 level in GC cells following the treatment of miR-326 mimic. ***p < 0.001 (n = 3) compared with miR-NC. (B, C) The proliferation and colony-forming was examined in GC cells when miR-326 was overexpressed. ***p < 0.001 (n = 3) compared with miR-NC. **p < 0.01 (n = 3) compared with miR-NC. (D) Transwell assay detection of the motility of GC cells which was received miR-326 mimic or miR-NC treatment. ***p < 0.001 (n = 3) compared with miR-NC. 100× magnification.

**Figure 7:**
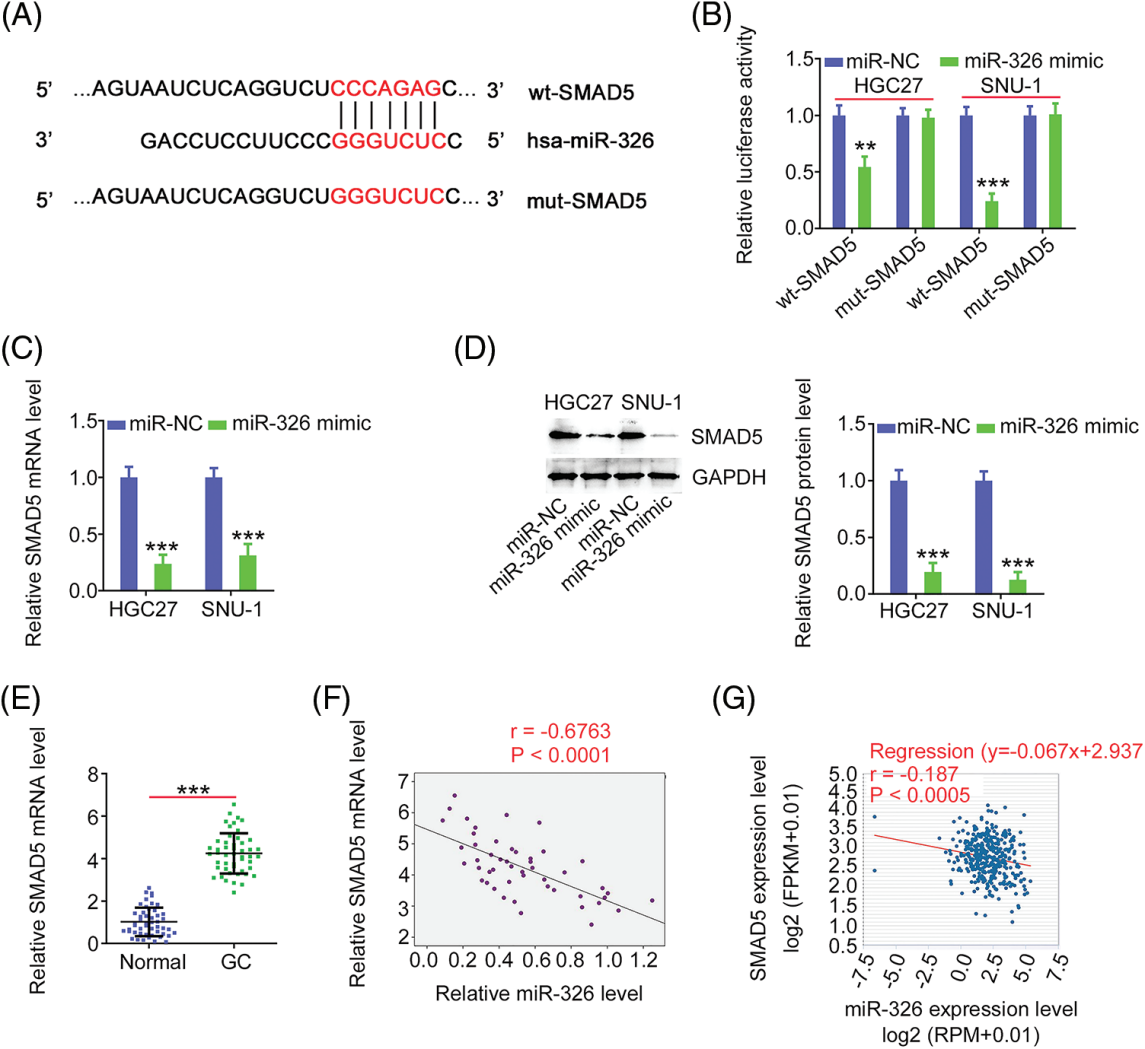
MiR-326 directly targets SMAD5 in GC. (A) Schematic representation of the binding sequences between miR-326 and SMAD5 3’-UTR. (B) The luciferase activity driven by wt-SMAD5 or mut-SMAD5 was detected in GC cells after miR-326 mimic or miR-NC transfection. ***p < 0.001 (n = 3) compared with miR-NC. **p < 0.01 (n = 3) compared with miR-NC. (C, D) SMAD5 level was detected in miR-326 overexpressed-GC cells. ***p < 0.001 (n = 3) compared with miR-NC. (E) Level of SMAD5 was detected in GC tissues. ***p < 0.001 (n = 3) compared with Normal. (F) Pearson’s correlation analysis was employed for illustrating the expression relation between miR-326 and SMAD5 in GC tissues from our own cohort. *p* < 0.001 (n = 3). (G) Pearson’s correlation analysis was employed for illustrating the expression relation between miR-326 and SMAD5 in GC tissues from TCGA database. *p* < 0.0005 (n = 3).

As miR-326 was expressed weakly, we overexpressed miR-326 in GC cells (Fig. 6A) and determined its effect on cancer progression. Significantly decreased cell proliferation (Fig. 6B) and colony formation (Fig. 6C) was observed in GC cells in presence of the miR-326 mimic. When miR-326 was upregulated, GC cells exhibited reduced migration and invasion (Fig. 6D). A bioinformatics prediction analysis identified SMAD5 as a putative conjugated target of miR-326 (Fig. 7A). As evidenced by the luciferase reporter assay, exogenous miR-326 expression resulted in a marked reduction of luciferase activity driven by wt-SMAD5; however, when the binding site was mutated, the regulatory activity was counteracted (Fig. 7B), implying binding between miR-326 and SMAD5. To further demonstrate the modulatory effect of miR-326 on SMAD5, SMAD5 levels were measured in miR-326-overexpressing GC cells, revealing that miR-326 downregulated SMAD5 levels (Figs. 7C and 7D). On the other hand, increased SMAD5 levels in GC tissues (Fig. 7E) have an inverse relationship with miR-326 expression (Fig. 7F), which is in agreement with the results obtained from the correlation analyses using GC samples from TCGA database (Fig. 7G). Collectively, miR-326 directly targets SMAD5 in GC.

### MiR-326/SMAD5 is required for the function of TMEM147-AS1 in GC

**Figure 8:**
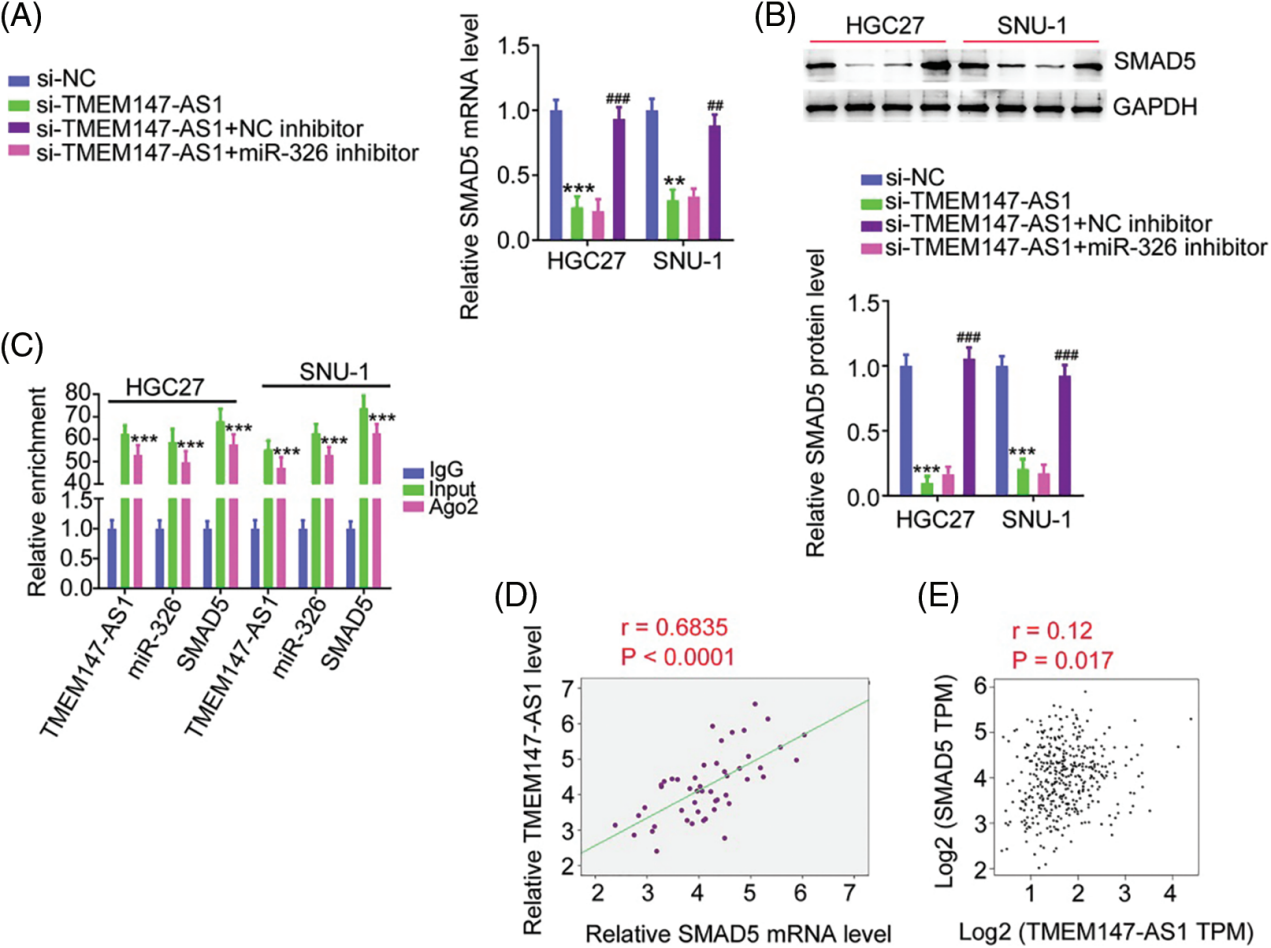
TMEM147-AS1 upregulates SMAD5 in GC through adjusting miR-326. (A, B) After transfecting miR-326 inhibitor into TMEM147-AS1-deficient cells, expression of SMAD5 was determined. ***p < 0.001 (n = 3) compared with si-NC. **p < 0.01 (n = 3) compared with si-NC. ^###^*p* < 0.001 (n = 3) compared with si-TMEM147-AS1+NC inhibitor. ^##^*p* < 0.01 (n = 3) compared with si-TMEM147-AS1+NC inhibitor. (C) RIP assay applying Ago2 antibody certified the enrichment of TMEM147-AS1, miR-326 and SMAD5 in GC cells. ***p < 0.001 (n = 3) compared with IgG. (D) In GC tissues from our own cohort, a positive relation was observed between SMAD5 and TMEM147-AS1. *p* < 0.0001 (n = 3). (E) In GC tissues from TCGA database, a positive relation was observed between SMAD5 and TMEM147-AS1. *p* = 0.017 (n = 3).

Based on the ceRNA theory, we determined the association among TMEM147-AS1, miR-326, and SMAD5. Depletion of TMEM147-AS1 suppressed SMAD5 levels in GC cells. Moreover, inhibiting miR-326 restored SMAD5 levels that were downregulated by si-TMEM147-AS1 (Figs. 8A and 8B). In addition, TMEM147-AS1, miR-326, and SMAD5 were all present in abundance in the products immunoprecipitated by the anti-Ago2 antibody (Fig. 8C). Furthermore, a correlation analysis revealed a positive relationship between TMEM147-AS1 and SMAD5 in GC tissues (Fig. 8D) and samples from TCGA database (Fig. 8E). Therefore, TMEM147-AS1 sponged miR-326 and thus affected SMAD5 expression in GC.

**Figure 9:**
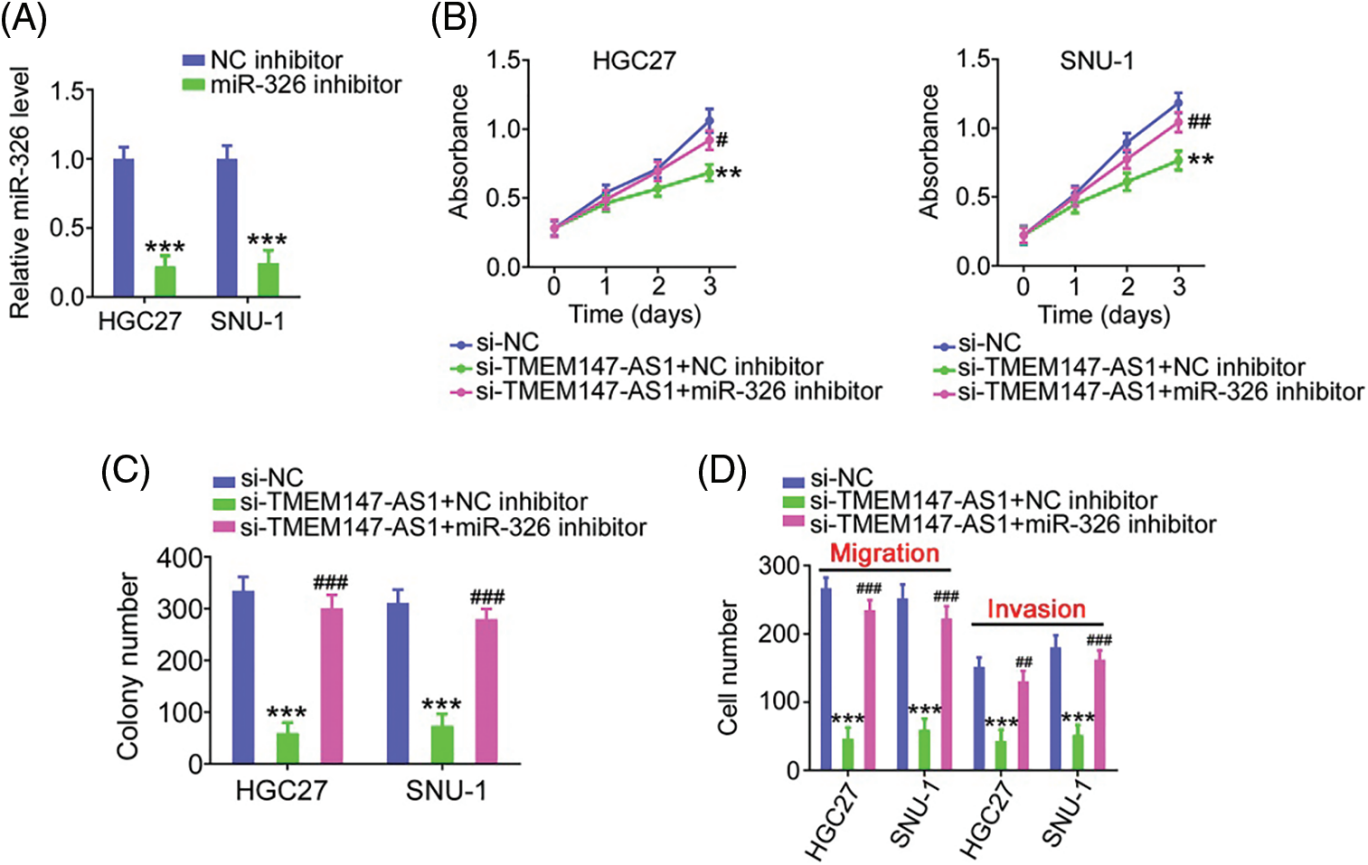
MiR-326 inhibitor reintroduction counteracts the effects of si-TMEM147-AS1 in GC cells. (A) qRT-PCR verified the decrease of miR-326 by miR-326 inhibitor. ***p < 0.001 (n = 3) compared with NC inhibitor. (B) Cell proliferation was examined in GC cells which were transfected with si-NC, si-TMEM147-AS1+miR-326 inhibitor, or si-TMEM147-AS1+NC inhibitor. **p < 0.01 (n = 3) compared with si-NC. ^##^*p* < 0.01 (n = 3) compared with si-TMEM147-AS1+NC inhibitor. ^#^*p* < 0.05 (n = 3) compared with si-TMEM147-AS1+NC inhibitor. (C) The colony formation was confirmed in TMEM147-AS1-silenced GC cells which were further received miR-326 inhibitor or NC inhibitor reintroduction. ***p < 0.001 (n = 3) compared with si-NC. ^###^*p* < 0.001 (n = 3) compared with si-TMEM147-AS1+NC inhibitor. (D) GC cells were transfected with si-NC, si-TMEM147-AS1+NC inhibitor or si-TMEM147-AS1+miR-326 inhibitor. The migratory and invasive capacities of GC cells were examined. ***p < 0.001 (n = 3) compared with si-NC. ^###^*p* < 0.001 (n = 3) compared with si-TMEM147-AS1+NC inhibitor. ^##^*p* < 0.01 (n = 3) compared with si-TMEM147-AS1+NC inhibitor.

**Figure 10:**
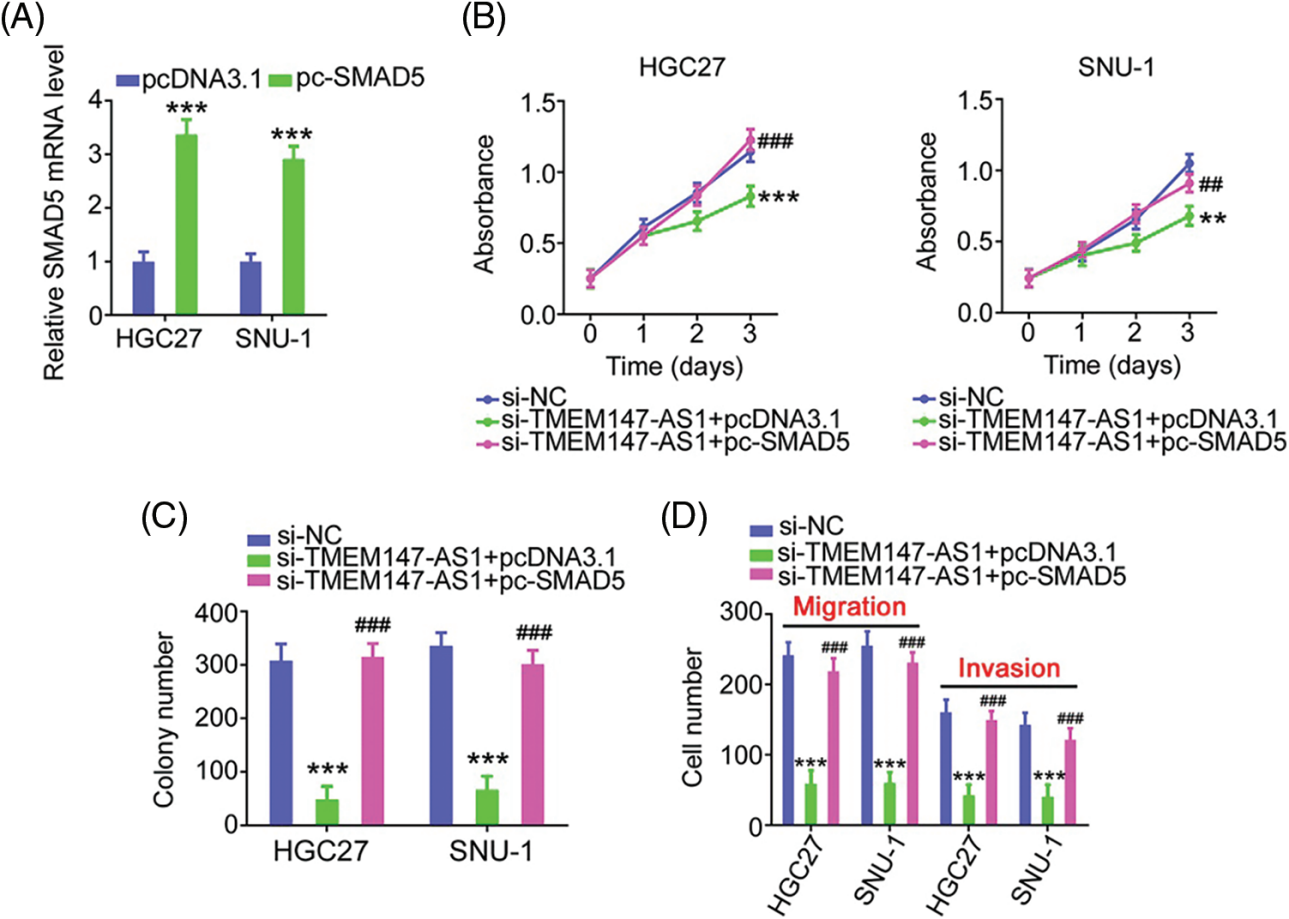
The modulatory influences of si-TMEM147-AS1 are offset by SMAD5 recovery. (A) qRT-PCR was applied for the measurement of SMAD5 in GC cells after being treated with pc-SMAD5 or pcDNA3.1. ***p < 0.001 (n = 3) compared with pcDNA3.1. (B, C) The proliferative and colony formation capacities were examined in GC cells following the treatment of si-NC, si-TMEM147-AS1+pcDNA3.1, or si-TMEM147-AS1+pc-SMAD5. ***p < 0.001 (n = 3) compared with si-NC. **p < 0.01 (n = 3) compared with si-NC. ^###^*p* < 0.001 (n = 3) compared with si-TMEM147-AS1+pcDNA3.1. ^##^*p* < 0.01 (n = 3) compared with si-TMEM147-AS1+pcDNA3.1. (D) After transfection with si-NC, si-TMEM147-AS1+pcDNA3.1, or si-TMEM147-AS1+pc-SMAD5, GC cell migration and invasion was determined. ***p < 0.001 (n = 3) compared with si-NC. ^###^*p* < 0.001 compared with si-TMEM147-AS1+pcDNA3.1.

Finally, we performed function-rescue experiments to determine whether the miR-326/SMAD5 axis is necessary for the action of si-TMEM147-AS1 in controlling the aggressiveness of GC. The transfection efficiency of miR-326 inhibitor in GC cells was examined utilizing qRT-PCR (Fig. 9A). si-TMEM147-AS1 suppressed cell proliferation and colony formation, which was offset by miR-326 inhibitor treatment (Figs. 9B and 9C). Furthermore, TMEM147-AS1 knockdown contributed to reduced cell migration and invasion capacities, which were restored by the inhibition of miR-326 (Fig. 9D). Simultaneously, qRT-PCR analysis corroborated the overexpression of SMAD5 by pc-SMAD5 (Fig. 10A). As expected, TMEM147-AS1 downregulation had repressive effects in GC growth (Figs. 10B and 10C) and metastasis (Fig. 10D); however, pc-SMAD5 rescued the phenotypes resulting from si-TMEM147-AS1 treatment. Therefore, the miR-326/SMAD5 axis plays an indispensable function in mediating the modulatory roles of TMEM147-AS1 in GC.

### TMEM147-AS1 ablation weakens tumor growth in vivo

**Figure 11:**
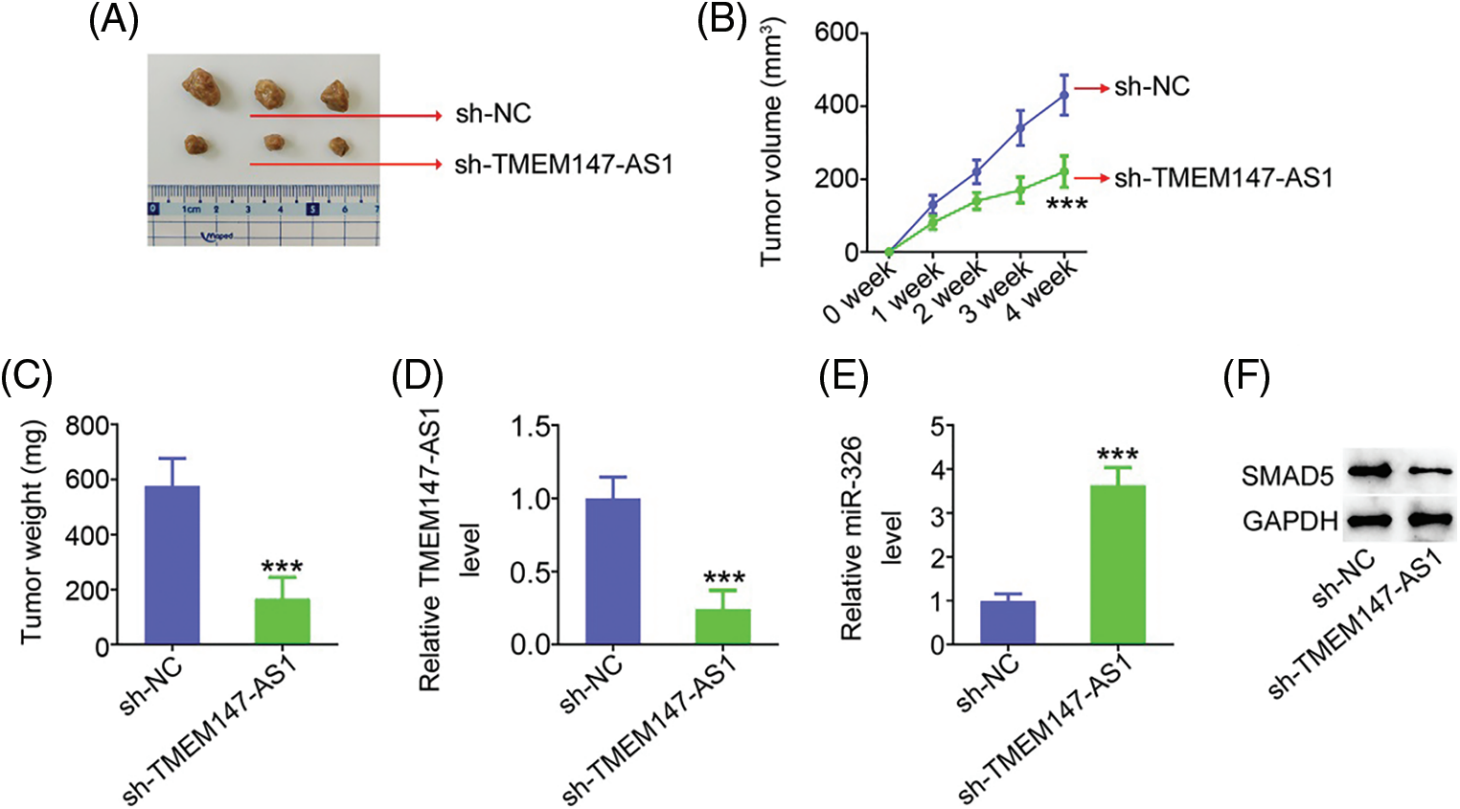
TMEM147-AS1 downregulation suppresses tumor growth *in vivo*. (A) Imaging of xenografts from sh-TMEM147-AS1 and sh-NC groups. (B) Tumor volumes were monitored weekly, after which were used for growth curves plotting. ***p < 0.001 (n = 3) compared with sh-NC. (C) Weight of the xenografts in sh-TMEM147-AS1 and sh-NC groups. ***p < 0.001 (n = 3) compared with sh-NC. (D) Total RNA was isolated, and subjected to the TMEM147-AS1 detection. ***p < 0.001 (n = 3) compared with sh-NC. (E) miR-326 expression in tumor xenografts was detected. ***p < 0.001 (n = 3) compared with sh-NC. (F) The protein level of SMAD5 was measured in xenografts from sh-TMEM147-AS1 and sh-NC groups.

Xenograft mice assay *in vivo* was actualized to illustrate the impact of TMEM147-AS1 on GC cell growth *in vivo*. HGC27 cells with stable sh-TMEM147-AS1 or sh-NC expression were subcutaneously injected into nude mice. As compared with sh-NC group, sh-TMEM147-AS1 injected-mice presented lowered tumor volume (Figs. 11A and 11B) and decreased tumor weight (Fig. 11C). After tumor excision, we executed molecular analysis, and verified that tumors originated from sh-TMEM147-AS1-transfected cells had a lower TMEM147-AS1 (Fig. 11D) and higher miR-326 (Fig. 11E) levels than those in sh-NC group. Additionally, SMAD5 protein expression was reduced in tumors that were acquired from sh-TMEM147-AS1 group (Fig. 11F). Altogether, inhibition of TMEM147-AS1 greatly restricts tumor growth *in vivo*.

## Discussion

As our understanding of cancer has progressed at the molecular level, growing evidence has indicated an important role of lncRNAs in GC [15,16]. Abnormal expression of lncRNAs is frequently observed in GC and considered an important driving force in GC progression [17]. Therefore, examining lncRNAs in GC may provide attractive options for the development of anticancer drugs. The present study illustrated the clinical relevance and specific roles of TMEM147-AS1 in GC. Notably, the mechanisms associated with TMEM147-AS1 were comprehensively exposed. Our results indicate that the loss of TMEM147-AS1 reduces the oncogenicity of GC by disrupting the miR-326/SMAD5 axis.

LncRNAs exhibit different expression profiles and features in GC. For example, CRART16 [18], LINC01270 [19] and HOXA10-AS [20] show high expression in GC and exert tumorigenic effects. In contrast, downregulation of CRYM-AS1 [21], MBNL1-AS1 [22], and HOXD-AS2 [23] have been demonstrated in GC and are capable of inhibiting cancer progression. However, little is known regarding the role of TMEM147-AS1 in GC. We evaluated TCGA data to identify a significant upregulation of TMEM147-AS1 in GC. Furthermore, qRT-PCR data supported the overexpression of TMEM147-AS1 in GC in our cohort. Interestingly, strong TMEM147-AS1 expression in GC was associated with a poor prognosis. With respect to the function of TMEM147-AS1, its interference resulted in reduced cell proliferation, colony-forming, migration, and invasion *in vitro*. Additionally, depletion of TMEM147-AS1 restricted the growth of GC cells *in vivo*. Therefore, TMEM147-AS1 may be an effective prognostic marker and treatment target for GC.

LncRNAs control cancer development and progression by attenuating gene expression through various mechanisms [24]. The actions of lncRNAs are primarily decided on the basis of their location. Nuclear lncRNAs function by regulating the RNA-binding of transcription factors or through epigenetic control, whereas in the cytoplasm, the ceRNA theory predicts a unique mechanism of action [25]. In the present study, we first determined the cellular location of TMEM147-AS1 and verified that it was localized in the cytoplasm. This suggests that TMEM147-AS1 affects the downstream targets through sequestration.

Using a bioinformatics prediction algorithm, we found that TMEM147-AS1 contains a complementary target site for miR-326. Subsequently, multiple experiments, including a luciferase reporter assay, RIP, and molecular detection, revealed that TMEM147-AS1 is a miR-326 sponge in GC. Because cytoplasmic lncRNAs can modulate the target of miRNAs, we inferred that TMEM147-AS1 may influence the direct target of miR-326. Subsequent mechanistic studies revealed that SMAD5 was targeted and inversely controlled by miR-326. Furthermore, TMEM147-AS1 sequestered miR-326 away from SMAD5; consequently, knocking down TMEM147-AS1 downregulated SMAD5 levels in GC cells. TMEM147-AS1, miR-326, and SMAD5 were also enriched by Ago2 in GC cells, suggesting their coexistence in the RNA-induced silencing complex. Thus, the three RNAs—TMEM147-AS1, miR-326, and SMAD5—constitute a newly identified ceRNA pathway in GC.

Accumulating studies have indicated an important role for miR-326 in the oncogenicity of human cancers [26–28]. In GC, the weak expression miR-326 has been confirmed [29,30] and a significant correlation with multiple aggressive clinicopathological characteristics is evident [29]. Functionally, the malignant phenotype of GC cells is tightly controlled by miR-326 [29–31]. In the present study, SMAD5 was demonstrated to be a functional effector of miR-326 in GC cells. Previous studies have reported that SMAD5 is a significant modulator of gastric carcinogenesis and progression [32]. Finally, by suppressing miR-326 or reintroducing SMAD5 into GC cells, we effectively reversed the attenuated biological behaviors that were caused by TMEM147-AS1 downregulation. Taken together, these observations offer sufficient evidence to indicate the involvement of TMEM147-AS1 in GC, which likely results from the alteration of the miR-326/SMAD5 axis.

This study explored the regulatory effect of TMEM147-AS1 depletion on GC cell phenotypes; however, gain-of-function experiments were not conducted. In addition, the influences of TMEM147-AS1 on GC cell metastasis *in vivo* were not explored. Moreover, the detailed mechanisms responsible for TMEM147-AS1 dysregulation were still unclear. In the near future, we will resolve these limitations.

TMEM147-AS1 is overexpressed in GC and exhibits cancer-promoting activities. Mechanistically, the inhibitory effects of TMEM147-AS1 knockdown on the malignant phenotype of GC were observed by scavenging miR-326 and consequently overexpressing SMAD5. Accordingly, targeting the TMEM147-AS1/miR-326/SMAD5 in GC may represent an efficient strategy for treating GC.

## Data Availability

The datasets used and/or analyzed during the current study are available from the corresponding author on reasonable request.
